# Assessment of the Neuroprotective and Stemness Properties of Human Wharton’s Jelly-Derived Mesenchymal Stem Cells under Variable (5% vs. 21%) Aerobic Conditions

**DOI:** 10.3390/cells10040717

**Published:** 2021-03-24

**Authors:** Ewelina Tomecka, Wioletta Lech, Marzena Zychowicz, Anna Sarnowska, Magdalena Murzyn, Tomasz Oldak, Krystyna Domanska-Janik, Leonora Buzanska, Natalia Rozwadowska

**Affiliations:** 1 Polish Stem Cell Bank, FamiCord Group, 00-867 Warsaw, Poland; ewelina.tomecka@pbkm.pl (E.T.); magdalena.murzyn@pbkm.pl (M.M.); tomasz.oldak@pbkm.pl (T.O.); 2 Department of Stem Cell Bioengineering, Mossakowski Medical Research Institute, Polish Academy of Sciences, 02-106 Warsaw, Poland; wlech@imdik.pan.pl (W.L.); mzychowicz@imdik.pan.pl (M.Z.); asarnowska@imdik.pan.pl (A.S.); kd-j@imdik.pan.pl (K.D.-J.); 3 Institute of Human Genetics, Polish Academy of Sciences, 60-479 Poznan, Poland; nrozwad@man.poznan.pl

**Keywords:** Wharton’s jelly mesenchymal stem cells, umbilical cord, oxygen conditions, secretory profile, neuroprotection

## Abstract

To optimise the culture conditions for human Wharton’s jelly-derived mesenchymal stem cells (hWJ-MSCs) intended for clinical use, we investigated ten different properties of these cells cultured under 21% (atmospheric) and 5% (physiological normoxia) oxygen concentrations. The obtained results indicate that 5% O_2_ has beneficial effects on the proliferation rate, clonogenicity, and slowdown of senescence of hWJ-MSCs; however, the oxygen level did not have an influence on the cell morphology, immunophenotype, or neuroprotective effect of the hWJ-MSCs. Nonetheless, the potential to differentiate into adipocytes, osteocytes, and chondrocytes was comparable under both oxygen conditions. However, spontaneous differentiation of hWJ-MSCs into neuronal lineages was observed and enhanced under atmospheric oxygen conditions. The cells relied more on mitochondrial respiration than glycolysis, regardless of the oxygen conditions. Based on these results, we can conclude that hWJ-MSCs could be effectively cultured and prepared under both oxygen conditions for cell-based therapy. However, the 5% oxygen level seemed to create a more balanced and appropriate environment for hWJ-MSCs.

## 1. Introduction

Mesenchymal stromal/stem cells (MSCs) are promising tools in regenerative therapy and other clinical applications. According to the position statement of the International Society for Cellular Therapy [1], MSCs are characterised by (1) adherence to plastic under standard culture conditions; (2) expression of the surface markers CD73, CD90, and CD105 in the absence of CD11b or CD14, CD19, CD34, CD45, CD79a, and HLA-DR; and (3) the ability to differentiate into adipocytes, osteocytes, and chondrocytes in vitro. Other key features of these cells are self-renewal; multipotency; high proliferative potential; and immunomodulatory, paracrine, and anti-inflammatory properties [2,3]. MSCs can be isolated from various types of adult tissue, namely, bone marrow, adipogenic tissue, and dental pulp and foetal tissue, namely, umbilical cord, umbilical cord blood, amniotic fluid, placenta, and Wharton’s jelly (WJ) [2,4,5,6]. The most common sources of mesenchymal stem cells used for clinical application are bone marrow and adipogenic tissue, which have some limitations [7,8,9,10,11]. For example, collection of these tissues is a painful and invasive procedure. Moreover, the proliferation ability and differentiation potential of human bone marrow-derived MSCs (hBM-MSCs) decrease with age [12,13].

A relevant source of MSCs is the umbilical cord matrix, or Wharton’s jelly, which is the embryonic mucous connective tissue between the amniotic epithelium and umbilical vessels. hWJ-MSCs also show similar characteristics to adult MSCs, as determined by the International Society for Cellular Therapy [1]. They exhibit a higher proliferation rate and differentiation capabilities and lower immunogenicity than adult tissue-derived MSCs [14,15,16,17,18]. hWJ-MSCs also have unique immunomodulatory effects [19]. Moreover, the isolation of hWJ-MSCs is easy and noninvasive [20]. In contrast to bone marrow, the umbilical cord is considered medical waste and is obtained/utilised with no harm to the patient. The expression level of neural/neuronal markers (Nestin, NF-200, GFAP) is higher in hWJ-MSCs than in hBM-MSCs [18], and mesenchymal stem cells derived from Wharton’s jelly exhibit neuroprotective properties, which were defined after indirect coculture of hWJ-MSCs with injured neuronal cells or tissue [21,22]. Due to these features of hWJ-MSCs, there is a growing interest in using these cells in cell-based therapies. Therefore, it is important to thoroughly investigate the properties of hWJ-MSCs.

Additionally, conventional in vitro cell cultures are carried out under atmospheric oxygen conditions (21% O_2_), which do not correspond to the in vivo situation. In living organisms, the average physiological oxygen concentration is much lower and varies from 2–9% O_2_ (“physiological normoxia”) [23,24,25] depending on the vascularization of tissue and its metabolic activity [26]. The oxygen level can be a significant environmental factor that may affect mesenchymal stem cell properties. Moreover, it has been hypothesised that 21% O_2_ culture conditions could lead to a reduction in MSCs’ therapeutic potential [27]. Therefore, it is important to determine the influence of different oxygen concentrations on the biological activity of MSCs to optimise their culture conditions before clinical application.

The role of different oxygen conditions in mesenchymal stem cell biology has been studied by other researchers, who investigated the influence of low oxygen tension (1–5%) on mesenchymal stem cells derived from bone marrow [27,28,29,30,31,32,33,34,35,36,37,38,39,40,41], adipose tissue [28,35,36,42,43,44,45,46,47,48,49], dental pulp [50,51,52], cord blood [53,54], the umbilical cord [45,55,56], and Wharton’s jelly [3,34,40,57,58]. However, studies on the effect of low oxygen levels on human WJ-MSCs were not comprehensive and have focused on a few cell properties. These works also reported contradictive results; thus, the impact of oxygen conditions on hWJ-MSCs has not been clarified.

The aim of the present work was to investigate and compare human Wharton’s jelly-derived MSCs cultured under atmospheric (21% O_2_) and low (5% O_2_) oxygen conditions. We analysed the different properties of hWJ-MSCs, such as the morphology, phenotype, proliferation ability, clonogenicity, senescence, mesodermal differentiation potential, secretory profile, metabolic activity, neural differentiation potential, and neuroprotective effects. The results of this work highlight the need to determine optimal oxygen culture conditions for the expansion of hWJ-MSCs intended for clinical application.

We would like to emphasise that the potential therapeutic application of MSCs requires careful verification of their properties prior to their transplantation. Standard tests based only on the expression of surface markers (CD73, CD90, CD105) and the capacity for mesodermal differentiation are insufficient. Depending on their subsequent use for treating specific neural disorders, the characteristics of mesenchymal stem cells should also include neural differentiation potential, neuroprotective properties, or the ability to secrete specific neurotrophins.

## 2. Materials and Methods

### 2.1. Isolation and Culture of Human WJ-MSCs

Umbilical cord (UC) fragments were collected from four human donors after caesarean section or natural delivery with the consent of mothers and approval by the Bioethics Committee. To minimise the risk of contamination, the umbilical cords were immediately transferred to the laboratory in a sterile transportation container filled with transport liquid consisting of 0.9% sodium chloride solution (Fresenius Kabi, Sevres, France) supplemented with antibiotic/antimycotic solution (Gibco, New York, NY, USA). After washing with fresh sterile transport liquid, the umbilical cords were cut into approximately 3 cm pieces, and the blood vessels were mechanically removed. Vessel-free Wharton’s jelly was sliced into 1–2 mm^3^ scraps, which were placed into 525 cm^2^ (triple layers, Falcon, Oxnard, CA, USA) culture flasks. The culture flasks were previously covered with MSC Attachment Solution Xeno Free (Biological Industries, Beit Ilaemek, Israel). For this purpose, culture surface of one culture flask was covered with 90 mL of attachment solution, prepared in a ratio of 1:100 in CTS DPBS (Gibco, New York, NY, USA), and incubated at 37 °C under a humidified atmosphere with 5% CO_2_ and 21% O_2_ for 30 min. After this time the culture flasks were rinsed with 90 mL of CTS DPBS (Gibco, New York, NY, USA). The explants were cultured with growth medium consisting of MSC NutriStem^®^ XF basal medium (Biological Industries, Beit Ilaemek, Israel) supplemented with MSC NutriStem^®^ XF Supplement Mix (Biological Industries, Beit Ilaemek, Israel) and 1% (*v*/*v*) antibiotic/antimycotic solution (Gibco, New York, NY, USA) at 37 °C under a humidified atmosphere with 5% CO_2_ and 21% O_2_. After up to 4 weeks (during this time culture medium was not exchange), the cultures were analysed for the presence of adherent, fibroblast-like cells. If nonadherent cells were present in the cultures, they were washed out. Subsequently, adherent cells isolated from Wharton’s jelly were detached with TrypLE Express Enzyme (Gibco, Denmark) and cryopreserved (passage 0) in 5% human serum albumin (CSL Behring, Margburg, Germany) containing 10% DMSO (WAK-Chemie Medical, Steinbach, Germany).

For all experiments, cells derived from four human donors (WJ-MSCs (1), WJ-MSCs (2), WJ-MSCs (3), and WJ-MSCs (4)) were used. First, the cells were thawed, centrifuged (112× *g*, 3 min) and seeded into 75 cm^2^ culture flasks at an initial density of 2 × 10^3^/cm^2^. The culture medium consisted of Dulbecco’s modified Eagle’s medium (DMEM, Macopharma, Mouvaux, France), 10% (*v*/*v*) human platelet lysate (Macopharma, Mouvaux, France), 2 U/mL heparin (Sigma-Aldrich, St. Louis, MO, USA), 1 mg/mL glucose (Sigma-Aldrich, St. Louis, MO, USA), and 1% (*v*/*v*) antibiotic/antimycotic solution (Gibco, New York, NY, USA). Before collection for subsequent passages, the cells were cultured to 70% confluence at 37 °C under atmospheric (21% O_2_ and 5% CO_2_) and “physiological normoxia” (5% O_2_ and 5% CO_2_) conditions. Experiments were performed with the use of cells at passages 1 to 5.

### 2.2. Flow Cytometry Analysis

An evaluation of the expression profile of surface markers was carried out using flow cytometry analysis. The cell phenotype was determined using a Human MSC Analysis Kit (Becton Dickinson, BD, Franklin Lakes, NJ, USA). For this purpose, the cells at passages 1–2 were detached with Accutase cell detachment solution (BD Bioscience, Franklin Lakes, NJ, USA) and resuspended in cold stain buffer (BD Pharmingen, Franklin Lakes, NJ, USA) at a minimal density of 1 × 10^6^ cells/mL. The WJ-derived cells were incubated with fluorochrome-conjugated (FITC, PerCP-Cy5.5, APC, PE) antibodies against CD105, CD90, and CD73 (mesenchymal cell surface markers) and CD11b, CD19, CD34, CD45, and HLA-DR (haematopoietic markers) for 30 min at room temperature in the dark. To exclude nonspecific binding, corresponding isotype antibodies (IgG1/IgG2) were used as a control. Cell analysis was performed using a FACSCalibur II flow cytometer and FACSDiva software (Becton Dickinson, Franklin Lakes, USA). The results are presented as the percentage of positive cells for suitable markers in relation to the isotype control.

### 2.3. Cell Proliferation Assays

The proliferation rate of the hWJ-MSCs was evaluated by a WST-1 assay (Roche, Mannheim, Germany) and expression analysis of the proliferation marker Ki67 (Abcam, Cambridge, MA, USA).

#### 2.3.1. WST-1 Assay

Quantitative analyses of cell proliferation were performed using a commercially available WST-1 assay. The principle of this test is based on the reduction of colourless tetrazolium salt to colourful formazan by cellular dehydrogenases. The amount of colourful product was then colourimetrically measured and directly correlated to the viable cell number. For this purpose, the cells at passages 3–5 were seeded into 96-well plates at a density of 2 × 10^3^ cells/cm^2^. For both analysed 7-day cultures (under 21% O_2_ and 5% O_2_), WST-1 reagent was added every day to the appropriate wells of 96-well plates at a volume ratio of 1:10 and incubated at 37 °C for 2 h. The absorbance was measured using a multiwell plate reader (FLUOstar Omega, BMG LABTECH, Ortenberg, Germany) at a wavelength of 420 nm. Based on the prepared standard curves, the obtained absorbance values were converted to the number of viable, metabolically active cells.

#### 2.3.2. Analysis of Ki67 Marker Expression

The proliferation ability of hWJ-MSCs cultured under 21% O_2_ and 5% O_2_ was evaluated by analysing the expression of the proliferation marker Ki67. The Ki67 protein is a nuclear antigen, and its expression is closely related to cell cycle activity. The expression of the Ki67 marker was determined by immunofluorescence staining analysis. For experiments, hWJ-MSCs at early (p1–p2) and late passages (p4–p5) were used. The cells were seeded into plates previously covered with poly-L-lysine (Sigma-Aldrich, St. Louis, MO, USA) coverslips placed in 24-well plates at a density of 2.5 × 10^3^ cells/cm^2^. When the cells reached 70% confluence, they were fixed with 4% paraformaldehyde solution (Sigma Aldrich, St. Louis, MO, USA) for 20 min at room temperature. To permeabilise the cell membrane, the cells were incubated with 0.1% Triton X-100 (Sigma Aldrich, St. Louis, MO, USA) for 15 min at room temperature. The nonspecific reaction was blocked with 10% goat serum (Sigma Aldrich, St. Louis, MO, USA) for 1 h at room temperature. The cells prepared in this way were incubated overnight at 4 °C with the primary rabbit polyclonal antibody anti-Ki67 (1:500, Abcam, Cambridge, MA, USA). Subsequently, the cells were incubated with Alexa Fluor 488 secondary antibody (1:1000, Thermo Fisher Scientific, Waltham, MA, USA) for 1 h at room temperature in the dark. Additionally, cell nuclei were stained with Hoechst dye (Sigma-Aldrich, St. Louis, MO, USA). The expression of the proliferation marker Ki67 was evaluated by microscopic observations. The labelled cells were counted from at least 6 independent images (~800 cells per image). The proliferation ability of hWJ-MSCs was expressed as the percentage of Ki67-positive cells to all cell nuclei.

### 2.4. Colony Forming Unit (CFU) Assay

For the colony forming unit assay, hWJ-MSCs at passage 2 were seeded into 6-well plates at a density of 10 cells/well. The cells were cultured for 14 days in complete medium under 21% O_2_ and 5% O_2_. To visualise the cell colonies, the cells were washed with PBS (Gibco, Bleiswijk, The Netherlands), fixed with 4% paraformaldehyde solution (Sigma Aldrich, St. Louis, MO, USA), and stained with 0.5% toluidine blue solution (Sigma-Aldrich, St. Louis, MO, USA). Colony forming potency was defined as the percentage of the colony number to the number of seeded cells.

### 2.5. Senescence-Associated β-Galactosidase Assay

A β-galactosidase assay was performed to evaluate cellular senescence. The activity of the β-galactosidase enzyme (cellular senescence biomarker) was detected with the use of a Senescence Cells Histochemical Staining Kit (Sigma-Aldrich, St. Louis, MO, USA). For the experiment, hWJ-MSCs at passage 5 were used. The cells were seeded into 6-well plates at a density of 2.5 × 10^3^ cells/cm^2^ and cultured until they reached 70% confluence. Subsequently, hWJ-MSCs were washed with PBS, fixed with fixation buffer, and then stained with staining solution according to the manufacturers’ protocol. The cells prepared in this way were incubated overnight at 37 °C, and then analysed by microscopic observations. The results of this study are presented as the percentage of blue-stained, senescent cells to the general number of analysed cells.

### 2.6. Mesodermal Differentiation Ability of hWJ-MSCs

The potential of human WJ-MSCs for differentiation into adipogenic, osteogenic, and chondrogenic lineages under 21% O_2_ and 5% O_2_ was evaluated. For the experiments, the cells at passage 3 were used. In the case of adipogenic and osteogenic differentiation, the cells were seeded into 24-well plates at an initial density of 2 × 10^3^ cells/cm^2^ in growth medium. After reaching 50–70% confluence, the growth medium was replaced by appropriate differentiation medium. Differentiation into cartilage tissue cells was performed by the hanging drop method (8 × 10^4^ cells/drop). After 1 h of incubation at 37 °C, the obtained cell aggregates were transferred into 24-well plates with appropriate differentiation medium.

Adipogenesis was induced by culturing hWJ-MSCs for 14 days in commercial adipogenic differentiation medium (Gibco, New York, NY, USA). Differentiation was confirmed by the staining method with Oil Red O (Sigma-Aldrich, St. Louis, MO, USA). Briefly, the cells were fixed with 4% paraformaldehyde solution (Sigma-Aldrich, St. Louis, MO, USA) for 30 min, washed 2 times with distilled water, and then with 60% isopropanol for 5 min (Merck, Darmstad, Germany). Then, the cells were stained for 5 min with Oil Red O (Sigma-Aldrich, St. Louis, MO, USA) to detect the presence of lipid droplets.

For the evaluation of osteogenesis, hWJ-MSCs were cultured for 21 days in commercial osteogenic differentiation medium (Gibco, New York, NY, USA). To verify osteogenic differentiation, the cells were fixed with 4% paraformaldehyde solution (Sigma-Aldrich, St. Louis, MO, USA) for 30 min, washed 2 times with distilled water, and stained with 2% Alizarin Red S (Sigma-Aldrich, St. Louis, MO, USA) for 3 min. Staining with Alizarin Red S allowed observation of the formation of calcium deposits.

Chondrogenic differentiation was performed using a commercial chondrogenic differentiation medium (Gibco, New York, NY, USA) for 14-day cultures. For verification of the differentiation of hWJ-MSCs into chondrocytes, the cells were fixed with 4% paraformaldehyde solution (Sigma Aldrich, St. Louis, MO, USA) for 30 min, washed 2 times with distilled water, and stained with 1% Alcian Blue (Sigma-Aldrich, St. Louis, MO, USA) for 30 min to observe protoglycans.

### 2.7. Analysis of hWJ-MSCs Secretome

Evaluation of the cytokine profile of Wharton’s jelly-derived MSCs cultured under different oxygen conditions (21% O_2_ and 5% O_2_) was carried out using ELISA and Luminex Multiplex assays (R&D Systems, Minneapolis, MN, USA). The secretion of the following factors was analysed: BDNF, INF-γ, TNF-α, IL-4, IL-6, IL-8, IL-10, IL-13, IL-17, VEGF, HGF, TGF-β, IGF-1, HLA-G, IDO, and PGE2. The following kits were used: LXSAHM-10 (for BDNF, CXCL8/IL-8, HGF, IFN-γ, IL-10, IL-17A, IL-4, IL-6, TNF-α, VEGF-A), DY6030-05 Human Indoleamine 2,3-dioxygenase/IDO DuoSet ELISA, DG100 Human IGF-I Quantikine ELISA Kit, DB100B Human TGF-beta 1 Quantikine ELISA Kit, KGE004B Prostaglandin E2 Parameter Assay Kit, D1300B Human IL-13 Quantikine ELISA Kit, and NBP2-62174 Human HLA G ELISA Kit. Culture supernatants at passages 1 and 5 were used for the experiment. When the cultures reached 70% confluence, the growth medium was replaced with serum-free medium to eliminate factors present in the platelet lysate. After the cells were incubated for 24 h with the new medium, the culture supernatants were harvested, centrifuged and thickened using filtering-thickening columns (Vivaspin 20, 5000 MWCO, Sartorius, Merck, Gloucestershire, United Kingdom). Thickened supernatants were cryopreserved at −80 °C until analysis. Quantitative analysis of TGF-β, IGF-1, IDO, and PGE2 was performed by ELISA (R&D Systems, Minneapolis, MN, USA), while other cytokines were analysed using a Luminex multiplex assay; both assays were performed according to the manufacturers’ protocol. Based on the absorbance measurements of protein quantity standards and tested samples (Bradford method), the obtained cytokine levels were calculated in relation to 1 µg of total protein. The results were expressed as pg/1 µg of total protein.

### 2.8. Determination of the Oxygen Consumption Rate (OCR) and Extracellular Acidification Rate (ECAR)

The metabolic potential of the hWJ-MSCs was evaluated using an Agilent Seahorse XF Energy Cell Phenotype Test Kit (Agilent Technologies, Santa Clara, CA, USA). This method allows one to simultaneously measure the two major energy-producing pathways of the cell, namely, mitochondrial respiration (determination of oxygen consumption rate (OCR)) and glycolysis (determination of extracellular acidification rate (ECAR)), under basal and induced stressed conditions. For this experiment, the cells at passage 2 were seeded into 8-well plates at a density of 5 × 10^3^ cells/well and incubated overnight under 21% O_2_ and 5% O_2_. Subsequently, the growth medium was replaced with assay medium consisting of Agilent Seahorse XF basal medium, 1 mM pyruvate, 2 mM glutamine, and 10 mM glucose and incubated with the cells for 1 h at 37 °C without CO_2_. Then, the OCR and ECAR were determined without the addition (baseline) and with the addition of electron transfer chain inhibitors (stressed). To generate stress conditions, the cells were treated with oligomycin (inhibitor of ATP synthase) and FCCP (mitochondrial uncoupling agent). The cells prepared in this way were analysed using an Agilent Seahorse XFe/XF Analyzer (Agilent Technologies, Santa Clara, CA, USA). Metabolic potential was expressed as the percentage of stressed OCR to baseline OCR and stressed ECAR to baseline ECAR.

### 2.9. Neural Differentiation Ability of hWJ-MSCs

Evaluation of the spontaneous differentiation potential of hWJ-MSCs towards neural progenitors (NG2, A2B5), glial (GFAP), and neuronal (β-tubIII, DCX, NF-200) cells was performed by immunofluorescence staining. For the experiment, the cells at passage 2 were seeded on poly-L-lysine-coated 24-well plates at a density of 2.5 × 10^3^ cells/cm^2^ and cultured under different oxygen conditions (21% O_2_ and 5% O_2_). After 48 h, the cells were fixed with 4% paraformaldehyde solution (Sigma Aldrich, St. Louis, MO, USA) for 20 min at room temperature and incubated with 0.1% Triton X-100 (Sigma Aldrich, St. Louis, MO, USA) for 15 min at room temperature to permeabilise cell membranes. The nonspecific reaction was blocked with 10% goat serum (Sigma-Aldrich, St. Louis, MO, USA) for 1 h at room temperature. The cells prepared in this way were incubated overnight at 4 °C with the following primary antibodies: polyclonal rabbit anti-NG2 chondroitin sulfate proteoglycan (1:300, Millipore, Burlington, MA, USA), mouse monoclonal anti-β-tubIII, IgG2b (1:1000, Sigma-Aldrich, St. Louis, MO, USA), polyclonal rabbit anti glial fibrillary acidic protein (GFAP, 1:500, Dako, Glostrup, Denmark), mouse monoclonal anti neurofilament 200, IgG1 (NF-200, 1:400, Sigma-Aldrich, St. Louis, MO, USA), mouse monoclonal anti A2B5, IgM (1:500, Millipore, Burlington, MA, USA), and polyclonal rabbit anti-doublecortin (DCX, 1:500, Cell Signaling Technology, Danvers, MA, USA). Subsequently, the antibodies were washed out and the cells were incubated with appropriate secondary antibodies conjugated with Alexa Fluor 488 or Alexa Fluor 546 fluorochromes (1:1000, Thermo Fisher Scientific, Waltham, MA, USA). Additionally, cell nuclei were stained with Hoechst dye (Sigma-Aldrich, St. Louis, MO, USA). Then, the labelled cells were analysed by microscopic observations.

### 2.10. Neuroprotective Properties of hWJ-MSCs

To analyse the neuroprotective abilities of hWJ-MSCs, coculture of human WJ-MSCs with organotypic rat hippocampal slices (obtained from 7-day-old rat pups) subjected to the transient oxygen-glucose deprivation (OGD) procedure was performed. Rat hippocampi were cut into 400 µm slices using a tissue chopper (McIlwan, Stoelting, Wood Dale, IL, USA) and placed onto the semipermeable membranes (Millicell CM, Millipore, Burlington, MA, USA) located in 6-well plates. A total of 900 µL of culture medium was added in each well. The composition of the medium was as follows: DMEM (Gibco, New York, NY, USA), horse serum (25%; Sigma-Aldrich, St. Louis, MO, USA), HEPES-Buffered Hanks Balanced Salt Solution (HHBSS, 25%; Gibco, New York, NY, USA), 1 M HEPES (Gibco, New York, NY, USA), 5 mg/mL glucose (Sigma-Aldrich, St. Louis, MO, USA), 1% amphotericin B, and 0.4% penicillin–streptomycin (Gibco, New York, NY, USA). Organotypic hippocampal slices were cultured at 35 °C. After 5 days of culture, when the concentration of the serum was lowered until it achieved a serum-free medium consisting of DMEM (Gibco, New York, NY, USA), HBSS (Gibco, New York, NY, USA), 1 M HEPES (Gibco, New York, NY, USA), glucose (Sigma-Aldrich, St. Louis, MO, USA), and antibiotics (Gibco, New York, NY, USA), the OGD procedure was performed. OGD causes early neuronal death in the CA1 region of the hippocampus. To mimic an ischemic injury, hippocampal slices were kept for 40 min at 35 °C in oxygen-free conditions that were prepared by placing the slices in an anaerobic chamber and saturating with 95% N_2_ and 5% CO_2_. The neuroprotective effect induced by hWJ-MSCs was determined as the ratio of neuronal death in the CA1 region of the hippocampus after OGD to neuronal death in the CA1 region of OGD-treated hippocampal slices after indirect coculture with hWJ-MSCs. For this purpose, human mesenchymal stem cells at passages 2–3 cultured under both oxygen conditions were seeded into 6-well plates and cocultured with OGD-treated hippocampal slices. After 24 h of coculture, the cells from the hippocampus were stained with 1.4 µg/mL propidium iodide (Sigma-Aldrich, St. Louis, MO, USA) to determine the number of dead cells in the CA1 region. Preparations were evaluated by microscopic observations.

### 2.11. Microscopic Observations

Analysis of the cell morphology, cellular senescence, and mesodermal differentiation potential of the hWJ-MSCs was carried out using a light microscope (Axio Vert. A1, Zeiss, Oberkochen, Germany). The expression of the Ki67 marker was analysed using an inverted fluorescence microscope (AxioVert 200, Zeiss, Oberkochen, Germany). All microscopes were coupled with a CCD camera. The neural differentiation ability and neuroprotective properties of the hWJ-MSCs were evaluated in the Laboratory of Advanced Microscopy Techniques, Mossakowski Medical Research Centre, Polish Academy of Sciences using a confocal microscope (LSM510, Zeiss, Oberkochen, Germany). For data acquisition and analysis, ZEN 2.3 (Zeiss) image analysis software was used.

### 2.12. Statistical Analysis

Each experiment was repeated independently 3–12 times. All obtained data are expressed as the mean ± standard deviation (SD). Statistical analyses were performed using GraphPad Prism 8 software (GraphPad Software, San Diego, CA, USA) and assessed using a one-way analysis of variance (ANOVA). Here, *p*-values of less than 0.05, 0.001, 0.0001, and 0.00001 were considered statistically significant.

## 3. Results

### 3.1. Morphology and Immunophenotype of hWJ-MSCs

**Figure 1:**
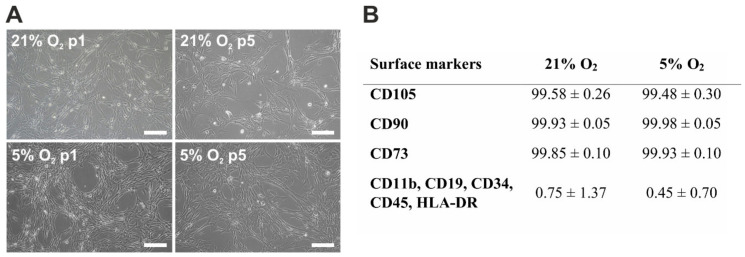
Morphology of the human Wharton’s jelly-derived mesenchymal stem cells (hWJ-MSCs) cultured under 21% O_2_ and 5% O_2_ (passages 1 and 5). (**A**) Phase contrast images for hWJ-MSC (3) donors are presented as a representative (scale bars: 100 µm). (**B**) Cell immunophenotype results are expressed as the mean ± SD of 4 investigated donors.

Human mesenchymal stem cells derived from Wharton’s jelly cultured under 21% O_2_ and 5% O_2_ were characterised by the typical morphology of MSCs (Figure 1A). Differences in morphology were not observed between the culture conditions or between passages and donors. The cells formed an adherent heterogeneous cell population in terms of the size and cytoplasm to nucleus ratio. They also exhibited fibroblast-like shapes during culture. The hWJ-MSCs cultured under both analysed oxygen conditions were positive for mesenchymal markers, such as CD105, CD90, and CD73, and negative for haematopoietic markers, such as CD11b, CD19, CD34, CD45, and HLA-DR (Figure 1B). Significant immunophenotypic differences were not observed between 21% O_2_ and 5% O_2_.

### 3.2. Cell Proliferation

**Figure 2:**
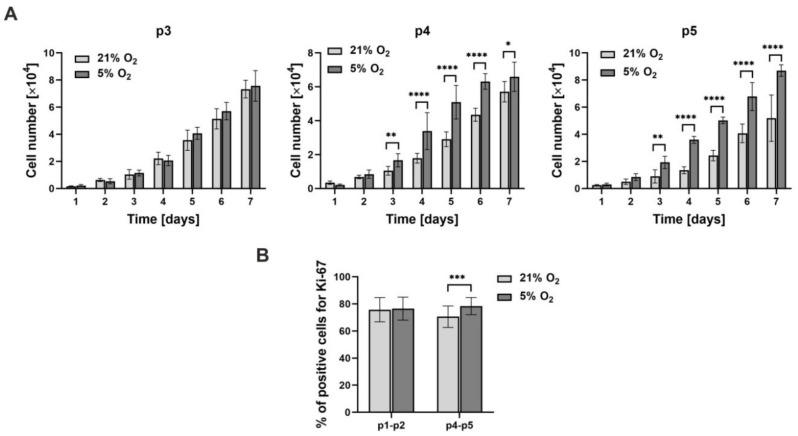
Results of proliferation of the hWJ-MSCs cultured under 21% O_2_ and 5% O_2_: (**A**) WST-1 assay (passages 3–5) and (**B**) expression of the Ki67 proliferation marker (passages 1–2 and 4–5). All results are expressed as the mean ± SD of 4 investigated donors at 6 replications (*n* = 24). The asterisks denote significant differences (* *p* < 0.05, ** *p* < 0.001, *** *p* < 0.0001, **** *p* < 0.00001) between 21% and 5% oxygen concentrations.

The proliferation of hWJ-MSCs was evaluated by two different methods: WST-1 assay and expression analysis of the proliferation marker Ki67. The results of the WST-1 assay (Figure 2A) showed that the number of cells cultured under different oxygen conditions increased each day of the culture for each investigated passage. Moreover, the cell proliferation rate was higher under 5% O_2_ than under 21% O_2_ for the cells at passages 4 and 5. At passage 3, a slightly higher cell number was observed under 5% O_2_ at days 5–7 of the cultures. However, the differences between 21% O_2_ and 5% O_2_ were not significant. For each following passage, the differences between the number of cells cultured under 21% and 5% oxygen levels were higher. In the case of passages 4 and 5, the cell proliferation rate was significantly higher under 5% O_2_ than under 21% O_2_ at days 3–7 of culture. At passage 5, the differences between the number of cells cultured under both oxygen conditions were the highest. Expression analyses of the Ki67 proliferation marker (Figure 2B) indicated that cells at passages 4 and 5 showed higher proliferation ability under 5% O_2_ than under 21% O_2_. At these passages, a significantly higher percentage of Ki67-positive cells under 5% O_2_ was observed. At passages 1–2, the cells showed a similar ability to proliferate under both investigated culture conditions.

### 3.3. Clonogenicity and Cellular Senescence of hWJ-MSCs

**Figure 3:**
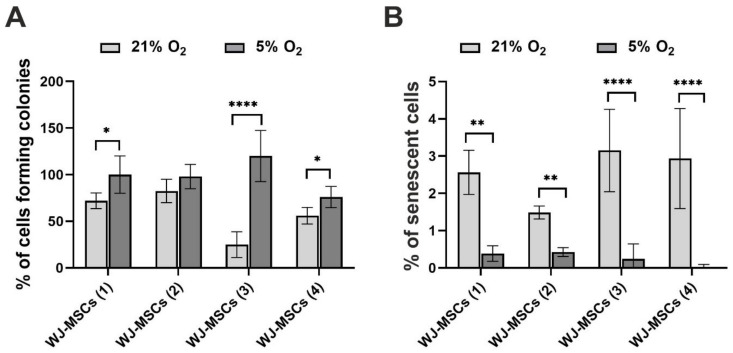
Results of the (**A**) colony forming unit (CFU passage 2) and (**B**) β-galactosidase assays (passage 5) for hWJ-MSCs cultured under 21% O_2_ and 5% O_2_. All results are expressed as the mean ± SD for the individual donors (*n* = 6 for the CFU assay and *n* = 12 for the β-galactosidase assay). The asterisks denote significant differences (* *p* < 0.05, ** *p* < 0.001, **** *p* < 0.00001) between the 21% and 5% oxygen concentrations.

Analysis of the colony forming potency (Figure 3A) indicated that cells cultured under 5% O_2_ exhibited higher clonogenic potential than cells cultured under 21% O_2_. For three of the investigated donors (WJ-MSCs (1), WJ-MSCs (3), and WJ-MSCs (4)), the differences between the two oxygen conditions were significant. In the case of one donor (WJ-MSCs (2)), the results were not significantly different. To evaluate cellular senescence, a β-galactosidase assay was performed. The results of this test are presented in Figure 3B and indicated that the hWJ-MSCs showed slight signs of cellular senescence. Moreover, the cultures under 5% O_2_ exhibited a significantly lower percentage of senescent cells than the cultures under atmospheric oxygen conditions. The same dependency was observed for all investigated cell donors.

### 3.4. Adipogenic, Osteogenic, and Chondrogenic Differentiation Potential of hWJ-MSCs

**Figure 4:**
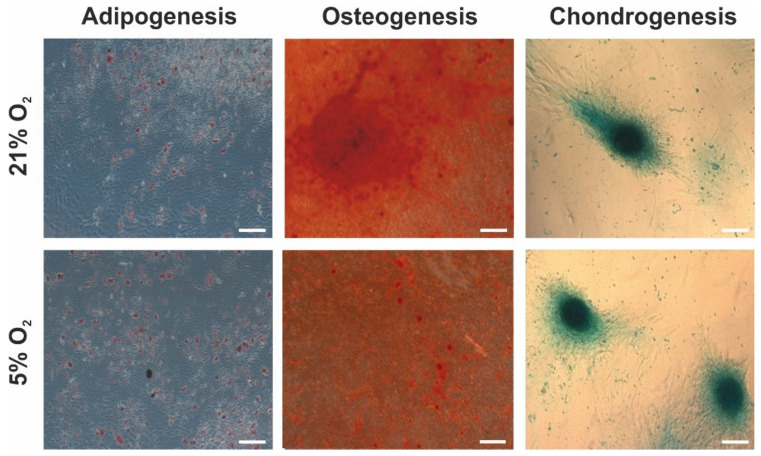
Multilineage differentiation potential of hWJ-MSCs cultured under 21% O_2_ and 5% O_2_ (passage 3). Phase contrast images of adipogenesis, osteogenesis, and chondrogenesis for WJ-MSC (1) donors are presented as representative (scale bars: 100 µm).

To evaluate the multilineage differentiation potential of hWJ-MSCs cultured under different oxygen conditions, adipogenesis, osteogenesis, and chondrogenesis were induced. The results (Figure 4) showed that the human mesenchymal stem cells derived from Wharton’s jelly exhibited the ability to differentiate into adipocytes, osteocytes, and chondrocytes under both 21% and 5% oxygen concentrations, and differences were not detected between the two culture conditions. Staining with Oil Red O indicated the presence of intracellular lipid droplets, which confirmed adipogenic differentiation. Alizarin Red S staining indicated the formation of calcium deposits, which are characteristic of osteocytes. Alcian Blue staining indicated the occurrence of proteoglycans, which confirmed the ability of the hWJ-MSCs to differentiate into chondrocytes. The same results were observed for all investigated donors.

### 3.5. Secretory Profile of hWJ-MSCs

**Figure 5:**
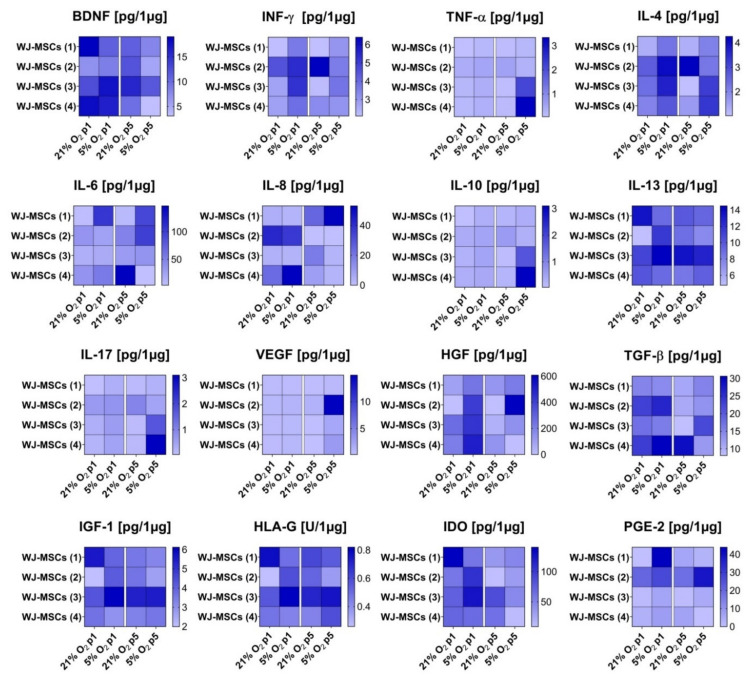
Secretory profile of hWJ-MSCs cultured under 21% O_2_ and 5% O_2_ (passages 1 and 5). The results are expressed as the mean for the individual donors (*n* = 4). The colour scale illustrates the relative expression of the analysed cytokines.

The influence of different oxygen conditions on cytokine secretion by hWJ-MSCs was also analysed. The analysis of the secretory profile, which is presented in Figure 5, indicated that 5% O_2_ promoted an increase in IFN-γ, IL-4, IL-6, HGF, TGF-β, and PGE-2 secretion for all or most investigated donors. The augmented secretion of these cytokines was observed for the cells at passage 1 as well as at passage 5. However, in the case of BDNF, a decrease in secretion under 5% oxygen was detected. This relationship was similar for most analysed donors and both investigated passages. For other analysed cytokines, depending on the cell donor, both an increase and a decrease in their secretion under 5% O_2_ were observed. However, due to large differences between donors, the dependence of the secretory profile of the hWJ-MSCs on the oxygen conditions of the cell culture could not be clarified. The results of the hWJ-MSC secretome also showed that cells isolated from all investigated donors secreted predominantly cytokines, such as HGF, IDO, and IL-6. In contrast, TNF-α, IL-10, IL-17, and VEGF secretion by hWJ-MSCs was at the lowest level.

### 3.6. Metabolic Potential of hWJ-MSCs

**Figure 6:**
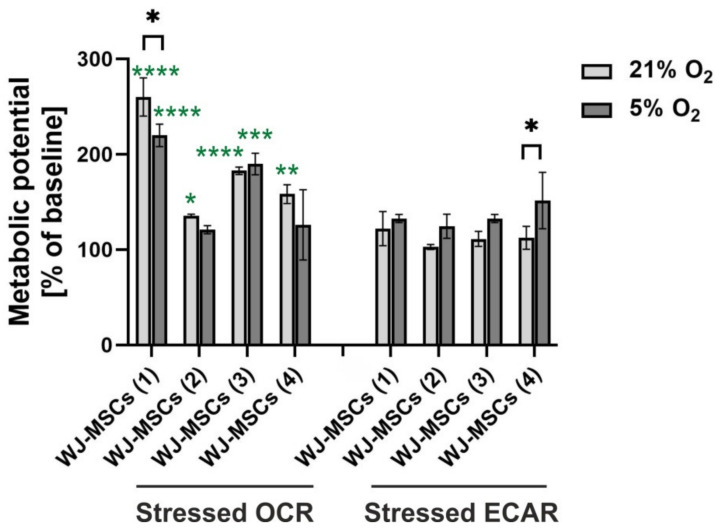
Metabolic potential of the hWJ-MSCs cultured under 21% O_2_ and 5% O_2_ (passage 2). The results are expressed as the mean ± SD for the individual donors (*n* = 4). The asterisks denote significant differences (* *p* < 0.05, ** *p* < 0.001, *** *p* < 0.0001, **** *p* < 0.00001) between 21% and 5% oxygen concentrations (black asterisks) as well as between stressed oxygen consumption rate (OCR) and extracellular acidification rate (ECAR) (green asterisks).

The analysis of the metabolic potential of the hWJ-MSCs cultured under 21% O_2_ and 5% O_2_ was based on the oxygen consumption rate (OCR) and extracellular acidification rate (ECAR). The results (Figure 6) indicated a significantly higher oxygen consumption rate than extracellular acidification rate under both oxygen conditions for most of the investigated donors (except for WJ-MSCs (2) and WJ-MSCs (4) donors under 5% O_2_). A higher OCR than ECAR suggests that the cells relied more on mitochondrial respiration than on glycolysis. Moreover, mitochondrial respiration was slightly higher under 21% O_2_ than under 5% O_2_ for most of the investigated donors (in the case of WJ-MSCs donor (1), the difference was significant). Opposite results were detected for glycolysis, which was slightly enhanced under 5% O_2_ (for WJ-MSC donor (4), the difference was significant).

### 3.7. Neural Differentiation Potential of hWJ-MSCs

**Figure 7:**
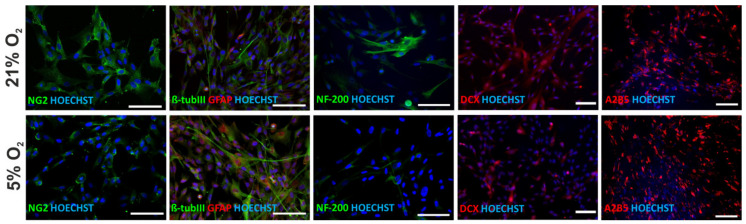
Neural differentiation potential of hWJ-MSCs cultured under 21% O_2_ and 5% O_2_ (passage 2). Fluorescent images showing the expression of markers of neural progenitors (NG2, A2B5), glial (GFAP), and neuronal (β-tubIII, DCX, NF-200) cells in Wharton’s jelly-derived human mesenchymal stem cells. Representative images of the cells isolated from WJ-MSC (2) donor are presented (scale bars: 100 µm).

The potential of human WJ-MSCs for differentiation into neural progenitors and glial and neuronal cells was evaluated. The results (Figure 7) showed that human mesenchymal stem cells derived from Wharton’s jelly expressed neural markers under both 21% and 5% oxygen conditions. The cells expressed markers of neural progenitors (NG2, A2B5), glia (GFAP), and neurons (β-tubIII, DCX, NF-200), which proves the ability of hWJ-MSCs to spontaneously differentiate into neurons. An immunocytochemical analysis performed on hWJ-MSCs isolated from four donors indicated a similar tendency to promote neuronal lineage differentiation (enhanced expression of NF-200 and DCX) of the hWJ-MSCs under atmospheric oxygen conditions. The expression of early neural progenitor markers (NG2 and A2B5) and glial marker (GFAP) was comparable under both oxygen conditions.

### 3.8. Neuroprotective Effect of hWJ-MSCs

**Figure 8:**
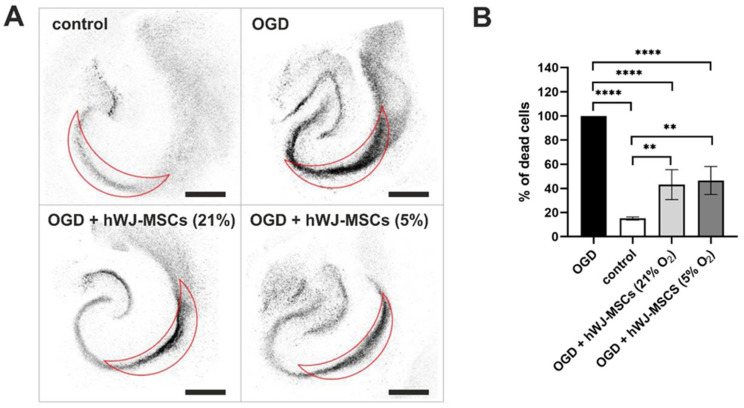
Neuroprotective effect of hWJ-MSCs cultured under 21% and 5% O_2_. (**A**) Confocal images of hippocampal slices for 4 investigated groups: control (without OGD), after OGD, after OGD and coculture with hWJ-MSCs from 21% O_2_, after OGD and coculture with hWJ-MSCs from 5% O_2_ (scale bars: 500 µm). The results for cells isolated from WJ-MSC (3) donor are presented as representative. (**B**) Quantitative evaluation of neuronal death in the CA1 region. The results are expressed as the mean ± SD of 4 investigated donors at 4 replications (*n* = 16). The asterisks denote significant differences (** *p* < 0.001, **** *p* < 0.00001) between the investigated groups.

To evaluate the neuroprotective properties of hWJ-MSCs, they were co-cultured with organotypic rat hippocampal slices subjected to the transient oxygen-glucose deprivation (OGD) procedure. The analysis of the results, presented in Figure 8, indicated that the hWJ-MSCs exhibited high protective potential in relation to OGD-treated nerve tissue. The percentage of dead cells in the CA1 region was significantly lower in hippocampal slices treated with OGD and cocultured with hWJ-MSCs than in hippocampal slices treated with OGD procedure and cultured without WJ-derived mesenchymal stem cells. No significant differences between the culture oxygen conditions were detected.

## 4. Discussion

Mesenchymal stromal/stem cells (MSCs) have regenerative properties and thus are increasingly used in cell-based therapies. However, the most commonly used sources of MSCs, such as bone marrow and adipogenic tissue, have some limitations [7,8]. MSCs isolated from Wharton’s jelly, which is considered medical waste and obtained with no harm to the patient, are becoming a popular source of cells for future applications in regenerative medicine. Therefore, it is important to thoroughly investigate the properties of these cells. Additionally, it is believed that oxygen conditions play an important role in stem cell physiology. Standard in vitro cell cultures under 21% O_2_ do not refer to a physiological stem cell niche. In vivo oxygen levels are much lower and range from 2% to 9%, thus representing ”physiological normoxia” [23,24,25]. In our study, we investigated and evaluated human Wharton’s jelly-derived MSCs cultured in vitro under different oxygen concentrations (21% O_2_ and 5% O_2_) and performed analysis of ten different hallmarks: morphology, phenotype, proliferation ability, clonogenicity, senescence, mesodermal differentiation potential, secretory profile, metabolic activity, neural differentiation potential, and neuroprotective effect. Previous studies on human WJ-MSCs’ activity under atmospheric and low oxygen levels [3,34,40,57,58,59,60,61,62,63,64,65,66] considered only a few cell properties. Moreover, the obtained results are often contradictory. 

Similar to previous works, the results of our experiments showed that oxygen levels do not influence the morphology [31,48,53,67] or phenotype of MSCs [27,34,35,42,46,48,49,50,51,52,53,54,57,60,61,65], and these features did not differ between cells cultured under 21% O_2_ and 5% O_2_. The hWJ-MSCs exhibited similar morphology and expressed surface antigens at similar levels under both investigated oxygen levels. The cells were positive for mesenchymal markers (CD105, CD90, CD73) and negative for haematopoietic markers (CD11b, CD19, CD34, CD45, HLA-DR). However, changes in cell morphology [34,50,65] and surface marker expression [62,67] in MSCs cultured under low oxygen concentrations have been reported. Nekanti et al. [34] indicated that hWJ-MSCs at early and late passages showed higher amounts of large and flattened cells under low oxygen relative to atmospheric oxygen. They explained that enlarging the cell surface probably promotes an increase in the oxygen diffusion rate to the cell. Different results were presented by Drela et al. [64], where hWJ-MSCs cultured under 5% O_2_ were smaller in size, had a round shape, and showed a tendency to form colonies compared to cells cultured under 21% O_2_. Similar morphological changes were indicated by Ahmed et al. [50]. Kwon et al. [67] observed in MSCs under 21% O_2_ enhanced expression of mesenchymal markers and reduced expression of haematopoietic markers under low oxygen. On the other hand, Majumdar et al. [62] detected reduced expression of mesenchymal markers under 21% O_2_.

Many studies proved the higher proliferation rate under low oxygen relative to atmospheric oxygen for different types of mesenchymal stem cells derived from: bone marrow (BM-MSCs) [31,36,38,62], adipose tissue (AD-MSCs) [36,44,45,48,49,68], cord blood (CB-MSCs) [28,45,53,54,55], amniotic fluid (AF-MSCs) [28], and Wharton’s jelly (WJ-MSCs) [3,34,40,58,60,65]. Our studies confirmed the previously reported results based on WST-1 assays and expression analyses of the Ki67 marker. They showed that the cell proliferation rate was significantly higher under 5% O_2_ than 21% O_2_ for the cells at passages 4 and 5. For the cells at passage 3, the differences between 5% O_2_ and 21% O_2_ were not significant. The analysis of the expression of the Ki67 proliferation marker indicated similar results. At passages 4 and 5, a significantly higher percentage of Ki67-positive cells (higher proliferation ability) was observed under 5% O_2_. In the case of early passages (1 and 2), the cells exhibited similar proliferative potential under both investigated culture conditions. In contrast, several works reported a lower proliferation rate of MSCs under low oxygen levels [35,42,44,46], while Roemeling-van Rhijn et al. reported that low oxygen does not influence the proliferation of AD-MSCs [47]. Such differences could result from the different concentrations of low oxygen and the duration of these conditions [23].

We also investigated the clonogenicity and cellular senescence of hWJ-MSCs. Analysis of the colony forming unit (CFU) assay results indicated that cells cultured under “physiological normoxia” exhibited higher clonogenic potential than cells cultured under atmospheric conditions. Such dependency was revealed for hWJ-MSCs [40,61] and for other mesenchymal stem cells [27,30,33,53,54,69]. However, Lee at el. [38] observed reduced colony forming efficiency of hBM-MSCs under low oxygen concentration.

Ageing cells show β-galactosidase enzyme activity, which is regarded to be a biomarker of cellular senescence [70,71]. Our results indicated that senescence (β-galactosidase activity) was inhibited under “physiological normoxia”. We observed a significantly lower percentage of senescent cells under 5% O_2_ in comparison to cultures under 21% O_2_, which suggests that “physiological normoxia” prevents senescence of hWJ-MSCs. Other studies on MSCs [53,67,72,73,74,75,76] confirmed our observations.

The multilineage differentiation potential of hWJ-MSCs is one of the reasons underlying the use of these cells in regenerative medicine [2]. In previous works, several groups demonstrated that low oxygen concentrations stimulated adipogenesis [27,40,43,45,47,63,77], osteogenesis [27,31,41,42,45,61,67,78], and chondrogenesis [30,39,40,41,46,49,61,69,79] of different types of MSCs. In most of these studies, the influence of 5% O_2_ on the differentiation potential of MSCs was investigated. Some of these works evaluated the differentiation ability of hWJ-MSCs under lower (e.g., 3%) oxygen conditions [40,61]. However, few studies have presented opposite results in which low oxygen levels (1–3%) suppress the differentiation of MSCs into adipogenic [37,49,79], osteogenic [37,40,46,49,61], and chondrogenic cells [37,80]. Furthermore, in other works [29,34,39,57], no significant differences in efficient mesodermal differentiation of MSCs between low (1–5%) and atmospheric oxygen levels were found. Similar to these results, our work also showed that human mesenchymal stem cells derived from Wharton’s jelly have comparable potential to differentiate into adipogenic, osteogenic, and chondrogenic lineages. No impact of oxygen level on the differentiation potential of the hWJ-MSCs was detected. Such heterogeneous results obtained in the abovementioned studies could be caused by the application of various levels of low oxygen (1–5%) tension and different exposure times to these conditions (72 h–30 days) [23]. It is worth noting the inconsistency across the publications in the terminology used to indicate oxygen level conditions. While the term “hypoxia” should be applied to describe oxygen concentrations lower than 1%, it also frequently refers to oxygen conditions, which are termed “physiological normoxia” in our study [23].

The immunomodulatory properties of MSCs are one of the main factors for using these cells in therapies [23]. MSCs secrete a variety of cytokines and growth factors that can influence tissue homeostasis and repair. According to several researchers [31,33,62], low oxygen increases the secretion level of several of these factors. Moreover, Teixeira et al. [59] indicated that low oxygen conditions led to an increased secretion profile of hWJ-MSCs compared to atmospheric conditions. They identified 104 factors for oxygen levels under 21%, 166 proteins for oxygen levels under 5%, and 81 proteins that were common for both oxygen conditions. In our work, we investigated the secretion of 16 factors. We demonstrated that 5% O_2_ promotes the growth of IFN-γ, IL-4, IL-6, HGF, TGF-β, and PGE-2 secretion. In the case of BDNF, a decrease in secretion under 5% oxygen was observed, which is similar to the work of Majumdar et al. [62]. In the work of Lech et al. [81], the mRNA expression of BDNF in hWJ-MSCs cultured in 3D hydrogel scaffolds was also decreased under 5% O_2_ in comparison to atmospheric conditions. However, due to the large differences between investigated donors, the dependence of the secretion of other factors on oxygen conditions could not be clarified. Detailed studies should be performed to investigate the influence of individual factors on hWJ-MSC properties.

Stem cells in their physiological environment (low oxygen conditions) rely more on glycolysis than on mitochondrial oxidative phosphorylation, although this relationship changes in favour of oxidative phosphorylation during their differentiation [23,82,83,84]. However, oxygen conditions significantly influence the process of respiration. When hWJ-MSCs were exposed to atmospheric oxygen conditions, glycolysis decreased in favour of mitochondrial respiration. Our analysis of the metabolic activity of the hWJ-MSCs confirmed that the cells cultured under 21% O_2_ relied more on mitochondrial respiration than on glycolysis. Moreover, mitochondrial respiration was slightly higher under 21% O_2_ than under 5% O_2_ for most of the investigated donors. Opposite results were detected for glycolysis, which was slightly enhanced under low oxygen concentrations. Lavrentieva et al. [55] and Dos Santos et al. [38] also observed higher consumption of glucose by MSCs under low oxygen than atmospheric conditions.

The spontaneous differentiation of hWJ-MSCs towards neural lineages observed in the current study confirmed our previous findings [18,22,64,66,81]. Drela et al. [18] described enhanced neuronal and glial differentiation of hWJ-MSCs as compared to hBM-MSCs cultured under 21% O_2_. In the current study early neural (NG2, A2B5), neuronal (β-Tubulin III, NF-200, DCX) and glial (GFAP) markers were expressed with no significant differences between oxygen conditions. However, an enhanced tendency for neuronal differentiation under 21% O_2_ was observed. This was in line with observed tendency of hWJ-MSCs neuronal lineage commitment in 21% O_2_ and 3D hydrogel scaffolds, but only at the protein level. The transcriptomic data previously obtained by our group [81] proved that lowered oxygen together with 3D hydrogel scaffolds together enhance the expression of neuronal markers. These results clearly show the importance of setting up appropriate in vitro biomimetic conditions (3D and oxygen tension) to obtain conclusive results regarding hWJ-MSC differentiation potential.

This unique feature of spontaneous differentiation of mesenchymal stem cells derived from afterbirth tissue towards neural cells favours the hypothesis about the regenerative potential of hWJ-MSCs; however, the ability of MSCs to differentiate into fully mature neurons and to functionally integrate with injured nerve tissue is still not proved.

The therapeutic effect of hWJ-MSCs is related mostly to their ability to secrete trophic and protective factors [22,85]. In this work, we confirmed this effect via the indirect (noncontact) coculture of these cells with damaged nervous tissue. Despite the lack of cell–cell contact, hWJ-MSCs significantly reduced apoptosis of neurons in the hippocampal CA1 region via secretion of neuroprotective factors in both tested oxygen conditions (21% O_2_ and 5% O_2_). A similar neuroprotective effect independent of the oxygen concentration was also observed by Lech et al. [81] for hWJ-MSCs cultured in 3D hydrogel scaffolds. Puig-Pijuan et al. also proved potential neuroprotective and antioxidants effects of WJ-MSCs on hippocampal cultures [86].

## 5. Conclusions

We characterised human mesenchymal stem cells derived from Wharton’s jelly (hWJ-MSCs) and evaluated the influence of different oxygen conditions on their properties. The results indicated that regardless of the oxygen level, hWJ-MSCs maintained the typical morphology and phenotype of mesenchymal stem cells according to the position statement of the International Society for Cellular Therapy [1]. Moreover, they have a higher proliferation rate and clonogenic potential under 5% O_2_ than under atmospheric conditions. The hWJ-MSCs displayed signs of cellular senescence during in vitro cultures; however, this process was reduced under “physiological normoxia”. The investigated cells have a similar ability to differentiate towards adipogenic, osteogenic, and chondrogenic lineages, and they present similar neuroprotective effects under both oxygen culture conditions. The ability of hWJ-MSCs to spontaneously differentiate into neuronal lineages was observed under 21% and 5% O_2_. Furthermore, cells rely more on mitochondrial respiration than on glycolysis under both oxygen conditions. However, the significance of these findings should be confirmed in preclinical trials conducted under defined conditions.

Based on our results, we can conclude that hWJ-MSCs are suitable as a cell source for application in regenerative medicine and display neuroprotective effect regardless of oxygen culture conditions. However, “physiological normoxia” favourably influences some regenerative properties of hWJ-MSCs and may enhance their therapeutic potential. Therefore, preconditioning cells under 5% O_2_ before transplantation might be beneficial. However, preclinical and clinical validation of our findings is required.

## Data Availability

The data presented in this study are available in the article.
